# Language attitudes and ethnic or cultural identity among ethnic minority university students in China: a systematic bilingual knowledge synthesis

**DOI:** 10.3389/fsoc.2026.1952915

**Published:** 2026-10-02

**Authors:** Wei Chen, Yok Fee Lee, Shikun Zheng, Ratna Roshida Binti Ab. Razak, Arfah Binti Ab. Majid

**Affiliations:** 1 Faculty of Human Ecology, Universiti Putra Malaysia, Serdang, Selangor, Malaysia; 2 School of Marxism, Zhaotong University, Zhaotong, China

**Keywords:** China, cultural identity, ethnic identity, ethnic minority university students, ethnolinguistic vitality, institutional recognition, language attitudes, systematic review

## Abstract

Research on ethnic minority university students in China frequently links language attitudes with ethnic or cultural identity, but its findings are difficult to accumulate because studies operationalize different constructs and answer different questions. This systematic bilingual knowledge synthesis examined what the evidence collectively establishes and which explanations remain provisional. Searches of Scopus, Web of Science Core Collection, and the China National Knowledge Infrastructure (CNKI) yielded 303 records; 25 reports (individual publications) representing 23 distinct studies (underlying empirical investigations or datasets) were included. Seven reports representing six studies supplied direct evidence, while 18 reports representing 17 studies supplied contextual evidence. Two reviewers independently screened records, extracted data, and appraised the included studies using the 2018 Mixed Methods Appraisal Tool (MMAT). Direct evidence showed a recurring association between language evaluation and ethnic or cultural identity, while direction, common magnitude, and causal ordering remain unresolved. Contextual evidence showed that heritage-language attachment can coexist with the instrumental value of Putonghua and English and identified opportunities for use and recognition as plausible explanations for variation in practice. The focal conclusion remained supported when it was based only on the six studies classified as direct evidence; contextual findings were not counted as confirmation. The review identifies a shortage of direct tests and limited comparability across constructs, designs, reports, and evidence roles as the main barriers to cumulative knowledge.

## 1 Introduction

Research on language attitudes and ethnic or cultural identity among ethnic minority university students in China has grown without producing a cumulative account of their relationship. Positive associations recur, but studies use language attitude to mean evaluation, proficiency, motivation, intention, or practice, while identity ranges from ethnic commitment to cultural belonging and interactional positioning. Findings that appear to address the same question therefore often estimate different relations.

The studies synthesized here concern China’s officially classified minzu categories, a state-administered ethnic classification system established through large-scale identification projects rather than a category that maps directly onto the distinction Western sociological scholarship draws between ethnicity and nationality (Mullaney, 2010). Minzu combines elements that Anglophone scholarship more often treats separately: in different institutional contexts, it marks cultural and linguistic distinctiveness as well as formal political recognition (Brubaker, 2004). This review therefore uses “ethnic minority” and “ethnic or cultural identity” as working translations of minzu-related constructs in the primary literature while retaining the conceptual specificity of the Chinese classification context.

The problem is compounded by differences in evidential role. Some studies directly measure both language evaluation and ethnic or cultural identity. Others examine language use, ideology, mobility, institutional provision, or identity negotiation and are better suited to explaining context than estimating the focal relationship. When these forms of evidence are combined without distinction, contextual plausibility can be mistaken for confirmation of a mechanism, and repeated reports from shared datasets can be mistaken for replication.

Publication language and disciplinary orientation further divide the knowledge base. Chinese-language surveys provide much of the large-sample evidence on attitudes, competence, and identity measures, whereas internationally indexed qualitative studies more often examine authenticity, linguistic legitimacy, mobility, and institutional recognition. These traditions are complementary, but they are not directly commensurable: they differ in constructs, designs, units of inference, and the claims they can sustain.

The field has produced substantial empirical material, but differences in constructs and evidential roles limit cumulative interpretation. The problem is especially visible in higher education, where heritage languages, Putonghua, and English carry different affective, social, academic, and occupational values. Institutional conditions may shape these relationships, although few studies measured them directly.

A recent systematic review of identity construction in China’s non-autonomous schools likewise identified linguistic challenges, cultural disparities, and institutional practices as recurrent themes (Yang et al., 2025). Its scope, however, centred on internal-migration schooling across educational levels and English-language records. The present review addresses a different question by combining Chinese- and English-language evidence on university students and separating studies that directly assess the language-evaluation–identity relationship from studies that provide contextual explanation.

This review asks what the literature can support about the relationship between language evaluation and ethnic or cultural identity once constructs, reports, independent studies, and evidence roles are separated. The analysis distinguishes direct associations from contextual interpretations, manages dataset dependence, and uses direct evidence for the central finding and contextual studies to interpret variation.

Stratifying the bilingual literature produces a narrower conclusion. Direct evidence supports a recurring but non-causal evaluation–identity association. Institutional recognition, mobility, digital participation, and language ideology remain propositions for longitudinal and multilevel research.

## 2 Methodology

### 2.1 Review design and protocol status

This systematic bilingual knowledge synthesis was reported with reference to the Preferred Reporting Items for Systematic Reviews and Meta-Analyses (PRISMA) 2020 statement (Page et al., 2021). The review grouped reports into independent studies and kept direct evidence separate from contextual evidence. Because the quantitative descriptive, qualitative, non-randomised, and mixed-methods studies used insufficiently comparable constructs, measures, and outcomes for meta-analysis, the review used structured narrative synthesis. The focal conclusion was then restated using only the studies classified as direct evidence. The protocol was not prospectively registered; dated search, screening, extraction, dependency, and appraisal records provide an audit trail, while retrospective protocol development remains a limitation.

### 2.2 Eligibility framework

The population criterion excluded broad university samples when findings for ethnic minority students could not be extracted separately. For example, Tang and Calafato (2024) studied 221 university students in southwest China, 65.6% of whom were Han, and reported latent profiles for the full sample. The article was therefore discussed as adjacent literature (Table 1).

**Table 1:** Operational eligibility criteria.

Domain	Included	Excluded
Publication	Peer-reviewed journal article reporting original empirical data	Thesis/dissertation, review, commentary, policy or conceptual essay, conference item, or non-empirical report
Population	Ethnic minority students in higher education in China, including undergraduate, preparatory, postgraduate, and vocational-college students; subgroup findings extractable	Studies limited to school, community, overseas, or post-university settings; mixed or majority Han university samples without separately extractable findings for ethnic minority students
Language construct	Language attitude; or language use, choice, ideology, maintenance, learning, or education explicitly connected to ethnic/cultural identity formation	Language learning or proficiency with no relevant attitude, identity, or mechanism
Identity construct	Ethnic, ethnocultural, group, cultural, or language and culture identity and closely specified belonging	National identity/community consciousness alone; general learner or multilingual identity without ethnic/cultural identity
Evidence role	Core direct evidence or explicitly justified contextual evidence	Mere co-occurrence of language and culture terminology without an analysable relation

### 2.3 Information sources and search construction

The last search was run on 24 July 2026. Scopus (Elsevier platform) returned 110 records, Web of Science Core Collection (Clarivate platform) returned 70, and the principal CNKI journal search returned 55. CNKI was one database searched twice: Reviewer 2 independently ran a supplementary search using the same four conceptual blocks and an expanded set of Chinese terms. This second CNKI run contributed 68 additional journal records to the combined pool. It also retrieved 30 theses or dissertations, which were set aside before pool construction because eligibility had already been restricted to peer-reviewed journal articles. Repeated Scopus and Web of Science exports served as reproducibility checks and were not counted again. The four search streams—Scopus, Web of Science, the principal CNKI run, and the supplementary CNKI run—contributed 303 records before deduplication (Figure 1).

**Figure 1:**
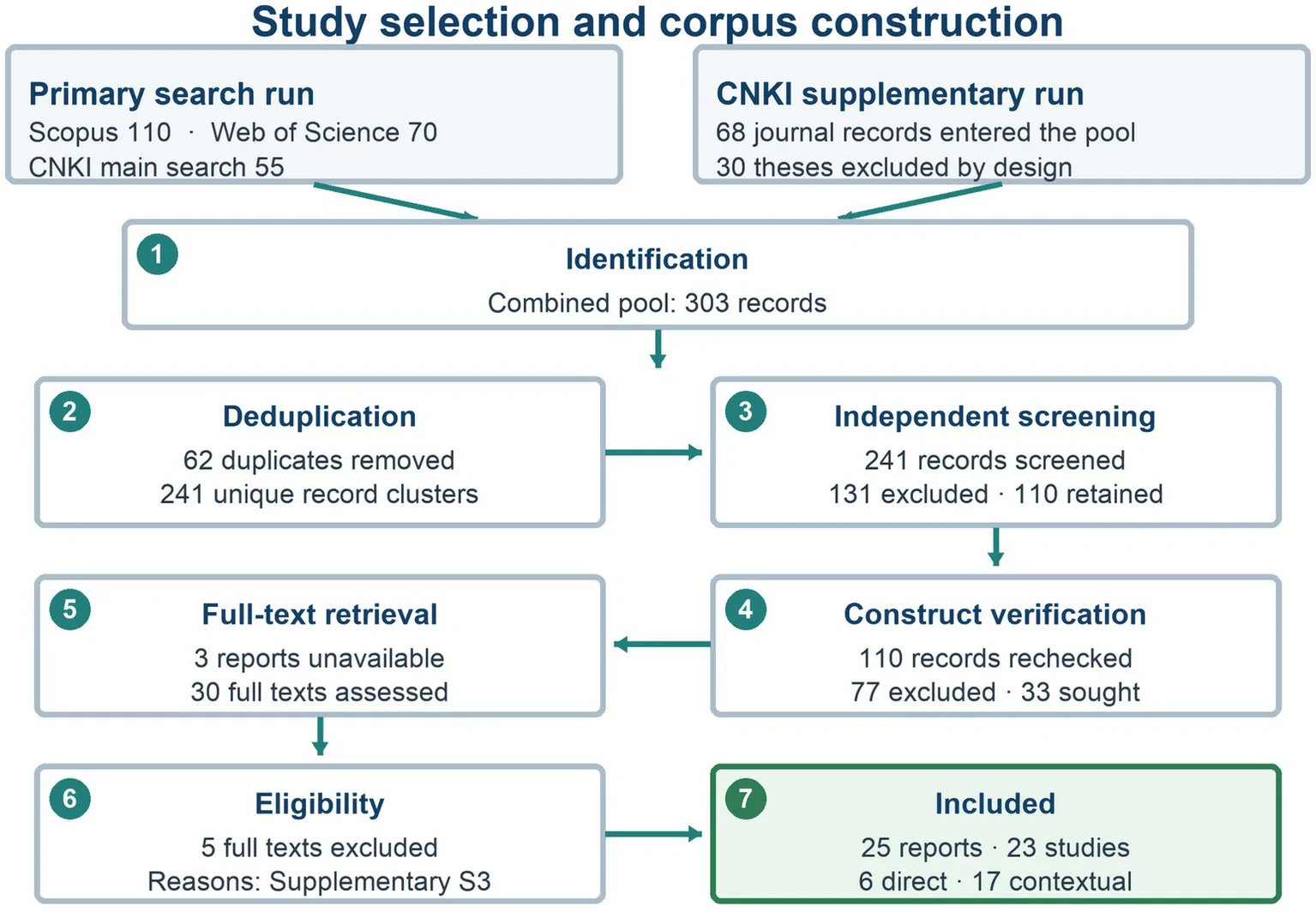
Study selection and corpus construction. Both reviewers independently completed the second construct-focused stage. Agreement (*κ* = 0.793) was calculated from their pre-consensus item-level decisions; disagreements were then reconciled record by record.

The search combined four blocks: the Chinese ethnic-minority context; higher education; language attitudes or adjacent language practices and ideologies; and ethnic, cultural, or language-and-culture identity. Scopus was searched in TITLE-ABS-KEY, Web of Science in Topic, and CNKI in the Topic field using Chinese equivalents. All searches were run directly in their respective database platforms, and the resulting records were retained as original platform exports. The full operational search strategies for all four search streams are reported in Supplementary Material.

### 2.4 Study selection and reviewer independence

Records were matched by persistent identifier and normalised title. Sixty-two duplicates were removed, reducing 303 records to 241 clusters. Reviewer 1 and Reviewer 2 independently screened all 241 titles and abstracts using a sensitivity-oriented three-category decision: proceed, exclude, or discuss. Three-category agreement was 83.4% (Cohen’s kappa = 0.706); binary agreement on proceed versus exclude was 90.0% (kappa = 0.802). After consensus adjudication, 110 records were provisionally retained and 131 excluded.

The first-stage rule was deliberately broad because potentially relevant studies sometimes discussed language use, ideology, or education without naming both focal constructs in the title or abstract. Before the second-stage decisions were made, the construct-focused criteria were fixed and applied independently by both reviewers to all 110 provisionally retained records. Agreement was calculated on the pre-consensus decisions (κ = 0.793). The reviewers then reconciled disagreements record by record, after which 77 records were excluded and 33 proceeded to retrieval.

Three of the 33 reports could not be retrieved and were therefore not included in the eligibility assessment or synthesis. Thirty full texts were assessed and five were excluded by consensus: one examined learner/multilingual identity rather than ethnic/cultural identity; one concerned national common-language identification and national-community consciousness only; one did not contain identifiable original empirical data; one was non-empirical and outside the identity boundary; and one combined university and high-school participants without separately extractable university findings. Audit identifiers and full exclusion reasons are reported in Supplementary Section S3. The final synthesis included 25 reports representing 23 distinct studies.

### 2.5 Data extraction and dependency management

Data were extracted independently by two reviewers using the same standardized form. The form recorded author, year, region, ethnic group or population, sample, study design, language construct, ethnic/cultural identity construct, principal findings, theoretical framing, and full-text evidence. The two forms were compared item by item, and discrepancies were resolved against the full texts. A report was defined as an individual publication, whereas a study was defined as the underlying empirical investigation or dataset and served as the unit of quality appraisal and synthesis. Reports based on the same dataset and participants were grouped at study level to avoid double-counting. Tang (2016a, 2016b) reported two waves from the same 30-person dataset and was treated as one study; Wang (2015a, 2015b) reported the same 10-college survey and was also treated as one study. Long and Duan (2012) and Long et al. (2011) came from the same project and shared one class but differed in fieldwork month, total sample, measures, and outcomes; they were retained as separate studies with a dependency flag. For presentation, the 23 distinct studies were assigned continuous identifiers S01–S23. Original audit identifiers are retained only in Supplementary Tables S1, S3.

### 2.6 Methodological quality appraisal

Two reviewers independently appraised the 23 distinct studies using the 2018 Mixed Methods Appraisal Tool (MMAT; Hong et al., 2018). For studies reported in more than one article, the reports were read together and one study-level judgment was made; duplicate reports did not contribute separate quality evidence. Each study was assigned to one design category and rated item by item as Yes, No, or Cannot tell. Category and item disagreements were resolved by documented consensus. The ratings were not summed into an overall score because aggregation would obscure design-specific weaknesses. Appraisal informed the strength of the synthesis rather than determining eligibility.

### 2.7 Synthesis strategy

The synthesis was organised around the review’s boundary-of-inference question. Core direct evidence comprised studies that independently operationalized language attitudes and ethnic or cultural identity and examined their relationship. Contextual evidence covered language use, learning, ideology, mobility, educational policy, institutional conditions, or identity interpretation without directly estimating that relationship. A comparison matrix recorded constructs, report dependency, design, setting, reported pattern, complicating evidence, and the MMAT limitation most relevant to interpretation. Findings were synthesized separately within the two evidence strata and then compared to identify contextual explanations for heterogeneity in the direct evidence; these explanations were treated as propositions for future testing. The focal conclusion was then restated using only the six core studies. The contextual studies were retained for interpretation and did not contribute confirmatory evidence. Interpretive confidence was judged from evidential directness, recurrence across independent studies, design limitations, and construct clarity; it was not a formal GRADE assessment.

## 3 Results

### 3.1 Corpus characteristics

The final synthesis included 25 reports representing 23 distinct studies published between 2011 and 2026. Seven reports, representing six distinct studies, provided core direct evidence, whereas 18 reports, representing 17 studies, were classified as contextual evidence. Kong (2023) was placed in the latter group because it measured language use, attitude, and learning motivation but did not independently operationalize ethnic or cultural identity; its identity claims are interpretive rather than observed outcomes. Six studies were conducted in Yunnan (e.g., Yang, 2015), five in Xinjiang, five in Beijing or other universities located outside the principal minority regions (e.g., He and Wang, 2016), three in Guangxi, and one each in Gansu, Inner Mongolia, and Fujian; one study reported a broader national sample without a single regional site. The regional pattern reflects where studies were conducted and published rather than the national distribution of language and identity processes. Supplementary Table S1 provides the publication-to-study mapping, study IDs, evidence roles, design, sample, and dependency status (Figure 2; Table 2).

**Figure 2:**
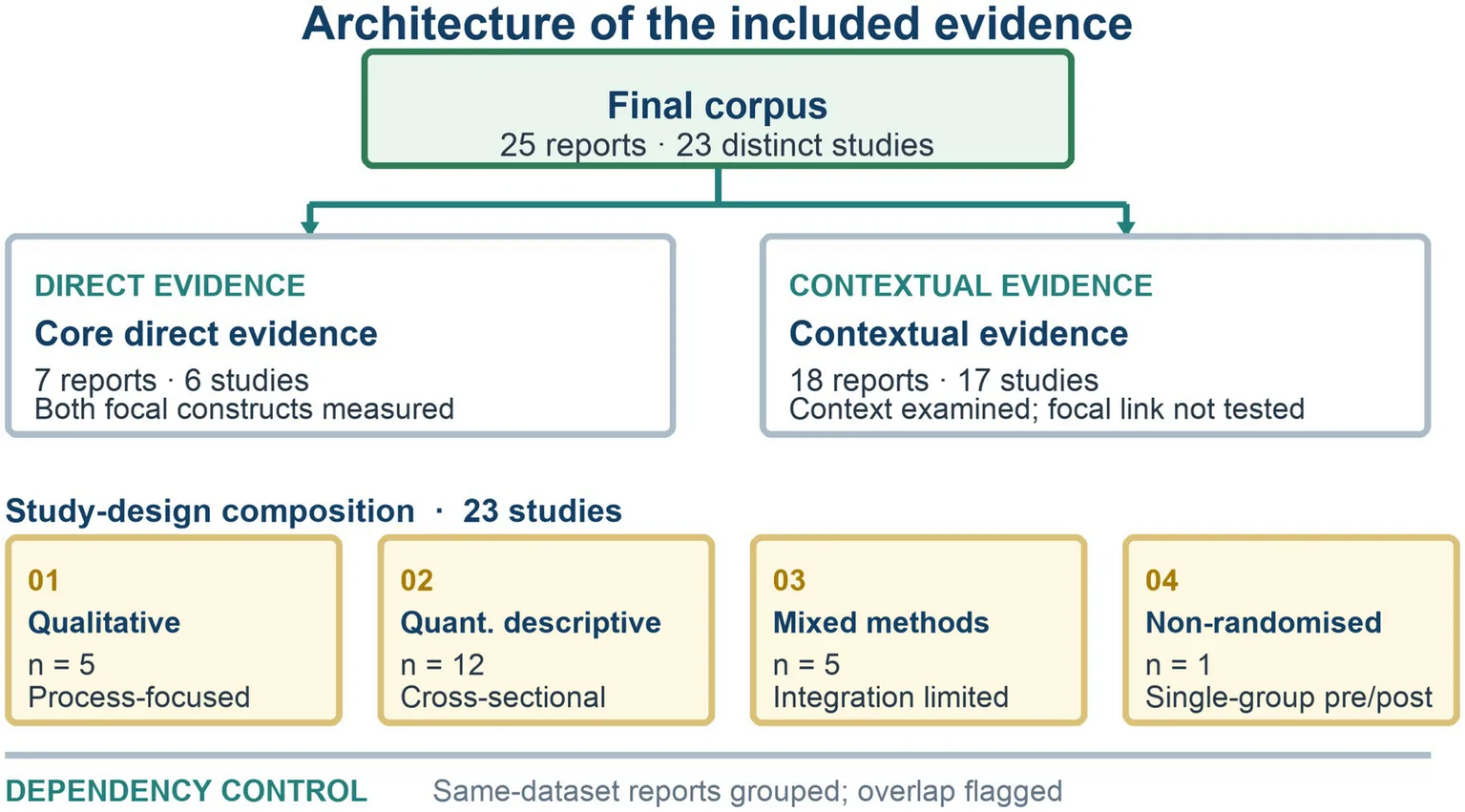
Architecture of the included evidence. Core and contextual evidence were synthesized separately before integration; report dependency was controlled at study level.

**Table 2:** Descriptive profile of the included evidence.

Characteristic	*n*	Interpretive significance
Reports/distinct studies	25/23	Two same-dataset report pairs combined; one partially overlapping pair flagged
Evidence role	6 core direct studies; 17 contextual studies	Contextual studies explain processes but are not treated as direct effect evidence
Quantitative descriptive	12	Predominantly cross-sectional self-report surveys
Qualitative	5	Ethnographic, narrative, and multi-source interview designs
Mixed methods	5	Complementary methods used, but integration frequently underreported
Quantitative non-randomised	1	Single-group pre/post study without a randomised comparator
Most represented regions	Yunnan 6; Xinjiang 5; Beijing/interior 5	Geographic and institutional concentration limits national generalisation

### 3.2 Methodological quality

All 23 studies passed the two MMAT screening questions. The qualitative subset was the strongest internally: each of the five studies showed alignment among the research question, data source, analysis, and interpretation. Their concentrated sites and purposive samples make them particularly useful for understanding processes of identity negotiation, authenticity, institutional recognition, and linguistic opportunity rather than estimating prevalence.

The 12 quantitative descriptive studies were more useful for mapping within-sample associations than for estimating population patterns. Analyses usually matched the stated questions and outcome data were generally complete. Sampling was the persistent weakness: relevance or representativeness was unclear in nine studies and inadequate in one, with only two receiving an affirmative judgment. Evidence for measurement validity was similarly uneven, being unclear in seven studies and adequate in five. Numerical precision in these reports should therefore not be confused with population-level certainty or causal leverage (Table 3).

**Table 3:** MMAT profile by study design.

Design	*n*	Principal strengths	Recurring limitations
Qualitative	5	All five criteria met; coherent sources, analysis, and interpretation	Institutionally concentrated samples limit prevalence claims
Quantitative descriptive	12	Questions and analyses usually aligned; outcome completeness generally adequate	Representativeness and measurement validity often unclear
Mixed methods	5	Use of complementary methods generally justified	Integration, divergence, and component quality insufficiently reported
Quantitative non-randomised	1	Provides longitudinal change information	No comparator; confounding not controlled; uncertainty concerning measurement or intervention fidelity

The mixed-methods evidence was less integrated than its design labels implied. Although all five studies offered some reason for combining methods, only two clearly integrated their components; one did not, and two provided insufficient information. None adequately addressed divergence between quantitative and qualitative findings. Component-level quality was also difficult to judge in three studies and inadequate in two. The sole non-randomised study followed participants at two points and retained complete outcomes; its small single-class sample, unaddressed confounding, uncertain measures, and unreported intervention fidelity support a suggestive interpretation of change rather than an intervention effect. Item-level MMAT judgments for all 23 studies are reported in Supplementary Table S2.

### 3.3 What the direct evidence establishes

The Results are organized by evidence role. This section presents the six direct-evidence studies; Sections 3.4–3.6 synthesize the contextual evidence, and Section 3.7 explains how direct and contextual evidence were kept separate and considers construct overlap.

The six studies classified as direct evidence formed a small, heterogeneous base. They comprised three cross-sectional surveys (*n* = 588, 1,048, and 100), a two-wave study of 30 participants reported in two articles, a Q methodology study with 46 Q-sort participants (39 retained for factor analysis), and a qualitative ethnography that included 168 background surveys, seven focus groups, and seven interviews. Given these differences in design, sample, and construct definition, the studies could not be pooled or treated as a uniform evidence base.

Across the six studies, evaluations of language covaried with ethnic or cultural identification. The three surveys reported positive associations among language attitude, competence, and ethnic identification, although their populations and construct definitions differed (Lin and Chen, 2022; Ma, 2017; Hu and Dong, 2017). Two reports from the same group of 30 Mongolian English majors recorded change across two measurement points (Tang, 2016a, 2016b). Without a comparison group, however, the observed change could not be attributed separately to educational exposure, maturation, or other experiences. Ethnographic and Q methodology findings indicated that students with similarly positive language evaluations could occupy different identity positions under different opportunities and language hierarchies (Liu, 2026; Mao et al., 2026). Recurrence across settings does not imply a common effect size.

Several ambiguities prevent stronger inference. In the core surveys, attitude, proficiency, motivation, and identification are not always kept analytically separate; an observed association may therefore reflect partially overlapping measures as well as a substantive relationship (Lin and Chen, 2022; Ma, 2017; Hu and Dong, 2017). Competence, exposure, family transmission, and institutional opportunity may also influence both evaluation and identity. Most evidence is cross-sectional, so regression “prediction” denotes conditional association rather than temporal influence. Even the two-wave Mongolian study lacks a comparison group and reports uncertain intervention fidelity, placing a clear ceiling on causal interpretation (Tang, 2016a, 2016b).

Language-related evaluations and ethnic or cultural identification often covary across survey, qualitative, Q-method, and two-wave evidence, but their direction remains unresolved (Lin and Chen, 2022; Ma, 2017; Liu, 2026; Mao et al., 2026). The meaning of this covariance varies with construct definition, measurement overlap, and the domains in which linguistic attachment can be enacted. The evidence supports a recurring association, not a universal attitude-to-identity pathway.

### 3.4 What contextual evidence adds: differentiated multilingual repertoires

Sections 3.4–3.6 draw on the contextual studies to examine multilingual repertoires, institutional conditions, mobility, digital settings, and language ideology.

Across Uyghur, Tibetan, Mongolian, Yi, Dai, She, Zhuang, and multi-ethnic samples, languages repeatedly carried different forms of value rather than occupying a single preference hierarchy. Uyghur surveys associated the mother tongue with affective attachment while assigning Putonghua educational and intergroup utility (Wang, 2017; Cui et al., 2015; Long et al., 2011). Studies in Yunnan, Guangxi, and Fujian likewise distinguished heritage-language affiliation from the institutional and mobility functions attached to Putonghua and English (Ling, 2020; Liu et al., 2021; Zhang and Wei, 2020). Because the studies used different domain measures, these convergent descriptive patterns were not pooled into a single hierarchy of language values.

This differentiated repertoire complicates a simple replacement narrative. In the Uyghur evidence, strong emotional orientation to the mother tongue coexisted with positive instrumental evaluation of Putonghua and heterogeneous orientations to English (Wang, 2017; Cui et al., 2015; Long et al., 2011). The Dai, multi-institution Yunnan, and Guangxi new-media studies similarly located language choice in family, campus, online, and anticipated employment domains rather than treating it as a single identity decision (Ling, 2020; Wang, 2019; Zhang and Wei, 2020). Q-method evidence from 46 minority preparatory students added another form of heterogeneity: participants clustered around distinct configurations of cultural security and language capital rather than one shared ordering (Mao et al., 2026).

The distinction between symbolic value and enacted practice is therefore central. Positive evaluation can coexist with limited language maintenance, while frequent Putonghua or English use can reflect institutional necessity rather than assimilation (Wang, 2017; Ling, 2020; Zhang and Wei, 2020). Heritage-language attachment may remain symbolically important where practical domains are restricted. The resulting analytical question is how students combine resources across audiences and which practices receive social and institutional recognition.

### 3.5 What contextual evidence adds: opportunities and recognition

The five qualitative studies made visible an explanatory pattern that survey correlations left implicit. Liu’s 18-week Yi ethnography combined background surveys with focus groups and interviews and showed how heritage-language attachment was negotiated under constrained opportunities for use (Liu, 2026). Tibetan evidence from a 15-month critical ethnography, a multi-source longitudinal qualitative study, and a 13-month campus ethnography linked identity positioning to curricular space, peer recognition, and authenticity boundaries rather than to attitude alone (Lhagyal, 2026; Wang and Chao, 2022; Yang, 2018). Uyghur language ethnography similarly showed that migration into interior universities reorganised the value and recognisability of multilingual resources (Guo and Gu, 2018).

Institutional recognition extends beyond formal policy. Across the Yi, Tibetan, and Uyghur qualitative studies, program design, teacher expectations, peer norms, assessment, student networks, and access to speakers operated as everyday recognition conditions (Liu, 2026; Yang, 2018; Lhagyal, 2026; Guo and Gu, 2018). Rhetorical celebration of diversity could therefore coexist with limited recurrent use. Underreported integration in the mixed-method evidence limited estimates of the relative contribution of particular institutional conditions (Wang, 2019).

These studies together offer a plausible account of why attitude and identity can be associated without forming a direct causal chain. Evaluation may remain private or symbolic when interlocutors, legitimate settings, or institutional support are limited; practice becomes identity-relevant when speakers and others recognise it as meaningful (Liu, 2026; Wang and Chao, 2022; Yang, 2018). Restricted opportunity may thus interrupt enactment without erasing attachment. Because no included study directly estimated institutional recognition as a mediator or moderator, this is a mechanism supported by qualitative convergence, not a tested effect.

### 3.6 Contextual variation across mobility, digital settings, and language ideology

Identity negotiation extended beyond classroom language choice. The Uyghur language ethnography followed 13 migrant university students through interviews, focus groups, and follow-up, showing how movement to interior universities changed the linguistic markets in which belonging was negotiated (Guo and Gu, 2018). Repeated interviews, WeChat contact, and observation with 11 Tibetan students likewise located identity positioning across home, campus, urban, and imagined professional settings (Wang and Chao, 2022). These small qualitative samples clarified process rather than the frequency of particular mobility trajectories.

Digital evidence was thinner. A Guangxi survey of 202 students identified online settings as additional domains of language choice and visibility, although its cross-sectional self-report design did not establish whether online practice protected or eroded ethnic-language identification (Zhang and Wei, 2020). Read alongside the interactional use of WeChat in the Tibetan qualitative study, it suggests that medium, audience, script, and purpose should be separated rather than treating “digital media” as a uniform influence (Wang and Chao, 2022).

Language ideology linked these settings by assigning value to speakers as well as to codes. Tibetan authenticity discourse showed how judgments of correctness and authenticity regulated legitimate group membership (Yang, 2018), while the multi-ethnic Q-method study identified different positions around cultural security and language capital (Mao et al., 2026). Together, these findings position recognition as an interpretive mechanism through which multilingual resources may be treated as capital in one setting and as deficit in another; its directional relationship with identity remains to be tested.

### 3.7 Separating direct and contextual evidence

The corpus uses language attitude, language identity, language-and-culture identification, proficiency, reported use, motivation, and investment with insufficient differentiation. Kong (2023), for example, infers ethnic and cultural identity from language use and attitude rather than measuring identity independently; the study is therefore contextual rather than direct evidence. Other reports place identity inside an attitude scale or treat language as one component of cultural identification (Zhang and Wei, 2020; Wang, 2015a, 2015b; Long and Duan, 2012; Lin and Lan, 2019). Measures bearing the same label can consequently represent different constructs, while measures with different labels can overlap.

Construct conflation explains both why the direct evidence cannot be pooled and why contextual studies cannot strengthen an association estimate. Table 4 offers a coding framework for future studies and reviews as well as a diagnosis of the present corpus. Several quantitative MMAT judgments identified insufficient item-level validity evidence (Kong, 2023; Zhang and Wei, 2020; Long and Duan, 2012). Once evaluation, capacity, intention, practice, and identification are separated, some apparent contradictions concern different relations rather than failed replication.

**Table 4:** Analytical distinctions required for future cumulative research.

Construct	What it captures	What it should not be used to infer automatically
Language evaluation	Affective, cognitive, or instrumental judgment of a language or variety	Actual use, competence, maintenance, or ethnic identification
Linguistic capacity	Self-reported or tested proficiency and repertoire	Attachment, willingness to use, or identity commitment
Situated practice	Use, choice, switching, and investment in specified domains	Stable preference or general identity strength
Ethnic/cultural identity	Belonging, exploration, commitment, cultural attachment, or boundary position	Language maintenance unless language is explicitly modelled
Institutional recognition/vitality	Perceived legitimacy, status, speaker access, and institutional support	An individual psychological trait or proven causal moderator

The focal conclusion was considered using only the six studies classified as direct evidence. Together, they indicated a recurring association between language evaluation and identity, but did not resolve its direction and often used overlapping constructs (Lin and Chen, 2022; Ma, 2017; Hu and Dong, 2017; Tang, 2016a, 2016b; Liu, 2026; Mao et al., 2026). The 17 contextual studies were used only to interpret possible variation related to institutional recognition, mobility, digital participation, and language ideology. They did not provide additional confirmation of the association. The focal conclusion therefore rests on direct evidence rather than on combining direct and contextual findings (Table 5).

**Table 5:** Claim-to-evidence matrix and interpretive confidence.

Synthesis claim	Evidence base	Confidence	Principal constraint	Permissible conclusion
Language evaluation and ethnic/cultural identity commonly covary	Six core studies across survey, Q-method, qualitative, and single-group longitudinal designs	Moderate	Construct overlap, cross-sectional dominance, and weak temporal ordering	A recurring association is supported; direction and causal magnitude are not.
Multilingual repertoires are functionally differentiated	Repeated contextual pattern across Uyghur, Tibetan, Yi, Dai, She, Zhuang, Mongolian, and multi-ethnic samples	Moderate	Domains and language functions were not measured consistently	Positive orientations to heritage language, Putonghua, and English can coexist.
Institutional recognition conditions enactment	Five qualitative studies plus contextual mixed-method evidence	Moderate (mechanism); low (effect)	No direct multilevel or moderation test	Institutional opportunity is a plausible boundary condition, not an estimated causal moderator.
Mobility and digital settings reposition identity	A small set of qualitative and new-media studies	Low to moderate	Few sites; medium, audience, and mobility are confounded	These settings alter available positions, but the direction of identity change is unresolved.
Construct conflation limits cumulative inference	Cross-design comparison of measures, labels, and MMAT measurement judgments	High	Primary reports often omit item-level validity evidence	A pooled language attitude and identity effect would be conceptually misleading.

Interpretive confidence is a transparent synthesis judgment based on directness, consistency, design limitations, and construct clarity; it is not a formal GRADE rating.

## 4 Discussion

### 4.1 Central synthesis: an association separated from its possible explanations

The six core studies support a recurring within-sample relationship between language evaluation and ethnic or cultural identity, but neither a common effect nor a directional claim (Section 3.3). Contextual studies help explain why similar evaluations may accompany different practices and identity positions: students encounter unequal domains of use, access to interlocutors, resources, mobility trajectories, and forms of recognition. These observations make institutional conditioning plausible without testing it. The distinction is therefore between what the corpus establishes and what it makes reasonable to investigate.

Figure 3 separates four domains that the primary literature often merges. Language evaluation concerns affective and instrumental judgment, distinctions blurred in several surveys (Ma, 2017; Hu and Dong, 2017). Opportunities concern the availability and legitimacy of domains for use, made visible in Yi, Tibetan, and Uyghur qualitative research (Liu, 2026; Yang, 2018; Guo and Gu, 2018). Situated practice concerns what students do across audiences and settings, including online and mobile contexts (Wang and Chao, 2022; Zhang and Wei, 2020). Identity concerns belonging, commitment, boundary negotiation, and recognition. Perceived ethnolinguistic vitality (Giles et al., 1977; Bourhis et al., 1981) and institutional recognition are retained as contextual variables to be measured, not as demonstrated mediators or moderators.

**Figure 3:**
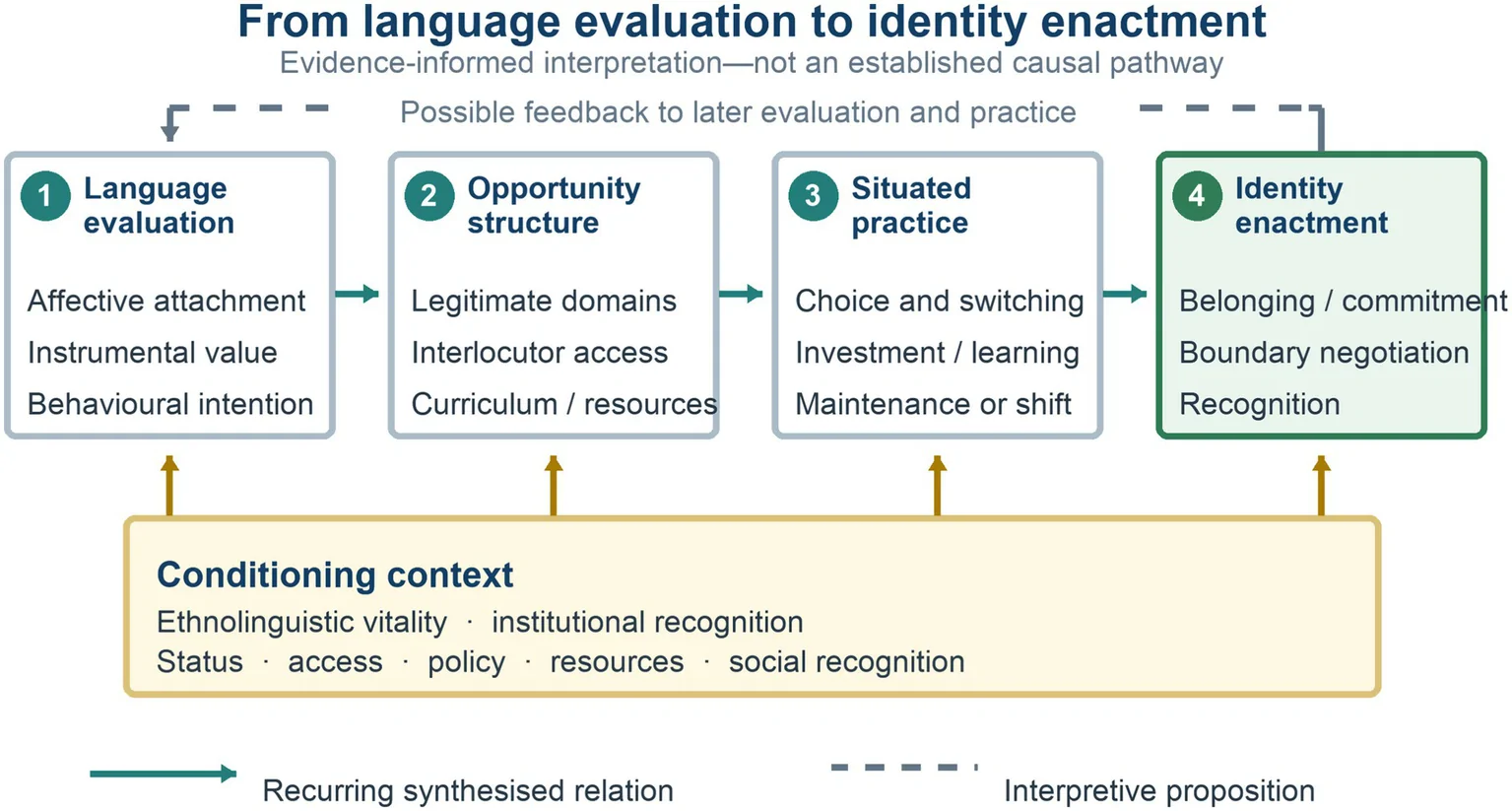
Evidence-informed interpretive framework. Arrows summarise recurring or plausible relationships and do not represent established causal effects. Perceived ethnolinguistic vitality and institutional recognition are contextual propositions requiring direct testing.

Survey and qualitative evidence contribute different kinds of knowledge. Surveys show that attitudes, competence, and identity often covary within particular samples (Lin and Chen, 2022; Ma, 2017; Hu and Dong, 2017), whereas ethnographic studies show how attachments are enacted under different conditions of opportunity and recognition (Liu, 2026; Yang, 2018; Lhagyal, 2026; Guo and Gu, 2018). Figure 3 brings these complementary contributions together: the evaluation–identity association is recurrent, the link between evaluation and practice is qualitatively plausible, and institutional conditioning is specified as a proposition for direct testing.

### 4.2 Contribution: a disciplined architecture for cumulative knowledge

This synthesis reorganises a heterogeneous corpus so that each claim is tied to the evidence that supports it. Independent study counts prevent repeated reports from appearing as replication. Separating core and contextual evidence keeps explanation distinct from direct association, while the distinction among evaluation, capacity, intention, practice, and identification shows why similarly labelled findings are not always comparable. The result is a recurring but non-causal evaluation–identity association and a set of contextual propositions that still require direct testing.

This contribution is complementary to the recent review by Yang et al. (2025). Whereas that review mapped how migration schooling shapes learner identity across educational levels, the present synthesis isolates the evidence for a specific relationship among university students, incorporates Chinese-language journal evidence, manages multiple reports from shared studies, and bases the focal conclusion on direct evidence while using contextual studies to interpret variation.

The review draws primarily on identity and investment (Norton, 2013; Darvin and Norton, 2015) to interpret the synthesized findings. This framework relates valued linguistic resources to opportunities, situated practice, and possible identity positions without assuming that attitudes directly cause identity. Three complementary lenses clarify narrower constructs: attitude and motivation research distinguishes affective from instrumental evaluation (Baker, 1992; Gardner, 1985); linguistic capital explains unequal institutional returns (Bourdieu, 1991); and language-policy analysis distinguishes belief, practice, and management (Spolsky, 2004). Together, these perspectives identify a question for future research: whether institutional opportunities and recognition alter the relationship between language evaluation, situated practice, and identity.

The qualitative evidence on boundary negotiation, authenticity discourse, and migration (Liu, 2026; Yang, 2018; Guo and Gu, 2018) is best read alongside constructivist accounts of ethnicity rather than through attitude theory alone. Barth's (1969) argument that ethnic boundaries are maintained through interaction, not enclosed cultural content, matches the corpus’s recurring finding that comparable attachments produce different identity positions depending on who recognizes a practice as legitimate. Brubaker's (2004) caution against treating ethnic groups as bounded, internally homogeneous actors is a relevant check on this corpus specifically: the studies synthesized here span minzu categories with markedly different histories of state relation and political salience, and the institutional-recognition pattern observed in one group’s qualitative evidence may not generalise to another. Cornell and Hartmann's (1998) account of ethnicity as continually constructed and reconstructed through institutional encounter is consistent with the corpus’s central finding that evaluation becomes identity-relevant under specific conditions of use and recognition.

Tang and Calafato (2024) provide a useful quantitative comparison. Their latent profile analysis of 221 university students in southwest China identified profiles that differed in ethnic composition, language proficiency and use, and plurilingual experience. The sample was 65.6% Han, and findings for eligible ethnic minority students could not be extracted separately, so the study fell outside this review’s population criterion. The profiles nonetheless complement the qualitative findings by showing that ethnic background and plurilingual experience may be patterned together.

The architecture may transfer beyond China where heritage, nationally dominant, and internationally valued languages carry unequal institutional returns. Transferability depends on whether studies preserve the same distinctions among evaluation, opportunity, practice, and identity; national location alone is not an explanation.

The bilingual corpus also has an uneven division of evidential labor. Chinese-language surveys supply much of the descriptive association evidence, whereas internationally indexed qualitative studies supply much of the contextual explanation. Neither can stand in for the other; they become cumulative only when interpreted within the limits of their designs.

### 4.3 Testing the contextual propositions

Only six of the 23 included studies measured language evaluation and ethnic or cultural identity separately within the same design. Most studies therefore cannot answer the review’s central question directly. A future direct test should measure three elements separately: students’ affective and instrumental evaluations of language; their ethnic or cultural identification; and the opportunities and recognition attached to language use. The measures should demonstrate discriminant validity so that any association is not an artefact of overlapping questionnaire items.

Different designs are needed for different questions. Longitudinal studies should follow the same students at several time points to determine whether change in language evaluation precedes, follows, or develops alongside change in identity. Multilevel studies should compare students across universities and measure curriculum, assessment, staffing, student organisations, access to speakers, and perceived legitimacy to test whether the evaluation–identity relationship differs across institutional settings. Comparative studies across minzu categories should sample institutional and political variation explicitly rather than assume that the same process operates in every group.

Mixed-methods studies should use the same participants or linked cases across components and should report where quantitative and qualitative findings agree or diverge. Each statistical model should match a specific question. Mediation asks whether opportunity or recognition helps explain the association between evaluation and identity; moderation asks whether the strength or direction of that association changes across levels of opportunity or recognition; and exposure analysis asks whether participation in a particular institutional setting is associated with the outcomes. These are different hypotheses and should be tested and reported separately. Qualitative comparison can then help explain why the observed patterns differ across institutions or groups.

### 4.4 Implications for higher education

Students’ positive attitudes toward a language do not by themselves show that their university supports its use. A clearer test is whether students can use the language repeatedly in consequential parts of university life—for example, in classes, assignments or assessment, student organizations, campus activities, digital spaces, and communication with teachers or peers. These practical opportunities show whether official recognition has become routine support.

The findings do not ask universities to choose between heritage languages, Putonghua, and English. A university can support Putonghua for study and wider participation, English for academic and mobility opportunities, and heritage languages as resources for knowledge, culture, and interaction. The practical question is whether students can use each language to take part in university life. Symbolic recognition alone is not enough.

### 4.5 Limitations

Several limitations qualify the synthesis. The protocol was not prospectively registered. The selected databases omitted several specialist education, psychology, and linguistics sources, and three potentially relevant reports could not be obtained in full text. The review should therefore be read as a critical synthesis of the available corpus, not an exhaustive account of all research on language attitudes and identity among ethnic minority students.

The corpus combines evidence from minzu categories with different histories of state relations, institutional treatment, and political salience, including Uyghur, Tibetan, Zhuang, Dai, and She populations. None of the included studies compared these groups directly, so the synthesis cannot determine whether institutional recognition operates in the same way across political contexts. Future comparative work should sample this variation explicitly and avoid treating minzu categories as interchangeable.

Further constraints include the retrospective operational clarification of the construct boundary, the restriction to Chinese- and English-language journal reports, uneven regional and institutional coverage, and one partially overlapping study pair. The clarified criteria were applied independently to all 110 provisionally retained records before the final retrieval set was agreed, but prospective registration would have provided a stronger safeguard. Most quantitative evidence is cross-sectional and self-reported, with limited evidence of representativeness or discriminant validity. Heterogeneity also ruled out meta-analysis, formal publication-bias assessment, and a quantitative certainty rating, while contextual evidence could not substitute for a direct test of the focal relationship.

These limitations set a firm ceiling on inference. The corpus cannot estimate national prevalence, establish that attitude causes identity, or compare language interventions. It can, however, identify why nominally similar findings are often incommensurable, prevent repeated publication from being mistaken for replication, and specify the institutional observations needed for stronger tests.

## 5 Conclusion

Direct evidence supports a recurring relationship between language evaluation and ethnic or cultural identity, while its direction, common magnitude, and causal ordering remain unresolved. Contextual studies describe differentiated multilingual repertoires and identify opportunities and recognition as plausible explanations for variation in practice that require direct testing.

Cumulative progress in this field depends less on adding broadly relevant studies than on designing direct tests and making constructs, designs, reports, and evidential roles comparable. Separating reports from studies, direct association from contextual explanation, and supported findings from propositions preserves what the current evidence can sustain while making the next empirical tests more precise.

## Data Availability

The original contributions presented in the study are included in the article/Supplementary material, further inquiries can be directed to the corresponding author.
