# Supplementary material for: Language attitudes and ethnic or cultural identity among ethnic minority university students in China: a systematic bilingual knowledge synthesis
Source: Front Sociol. 2026 Oct 2;11:1952915. doi: 10.3389/fsoc.2026.1952915 (PMC13671949; doi:10.3389/fsoc.2026.1952915)
Supplement: Supplementary file 1 [file Supplementary_file_1.DOCX]

**Supplementary Material**

This file accompanies the main manuscript and reports the study-level records referenced there as Supplementary Tables S1–S2 and Supplementary Materials S3–S4.

Contents: Table S1, publication-to-study mapping and characteristics; Table S2, MMAT 2018 judgments; S3, full-text exclusions; S4, complete database search strategies.

# Table S1. Publication-to-study mapping and characteristics of the 23 included distinct studies

Reports based on the same dataset are displayed under one Study ID; the partially overlapping pair is retained as separate studies with a dependency flag.

| **Study ID** | **Original audit ID(s)** | **Publication(s)** | **Evidence role** | **Region / population** | **Design / sample** | **Dependency** |
| --- | --- | --- | --- | --- | --- | --- |
| S01 | C025 | Liu, J. (2026). Constrained but creative: Yi undergraduates' perspectives on heritage language and culture in China. Language and Intercultural Communication. https://doi.org/10.1080/14708477.2026.2660962 | Direct | Yunnan; Yi undergraduates | Qualitative ethnography; 168 background surveys; 7 focus groups; 7 interviews; 18-week fieldwork | Independent report |
| S02 | C089 | Mao, F., Zhu, Y., Ling, Z., Qian, J., & Su, Z. (2026). Multilingual transitions and language attitudes: Cultural security and language capital among Chinese ethnic minority students. International Journal of Multilingualism. https://doi.org/10.1080/14790718.2026.2642401 | Direct | China / multi-site; Ethnic minority preparatory students | Q methodology + interviews; 46 Q-sort participants; 39 retained for factor analysis | Independent report |
| S03 | C135 | Kong, W. (2023). Exploring Putonghua course teaching in higher education based on a language survey [in Chinese]. Data, (2), 182–185. | Contextual | Yunnan; Ethnic minority university students | Quantitative descriptive; 29 universities; 1,168 valid questionnaires | Independent report |
| S04 | C137 | Lin, L., & Chen, Y. (2022). Ethnic identity, language proficiency, and language attitudes among Zhuang university students [in Chinese]. Comparative Study of Cultural Innovation, 6(23), 29–32. | Direct | Guangxi; Zhuang university students | Quantitative descriptive; 588 valid questionnaires | Independent report |
| S05 | C153 | Ma, Z. (2017). The relationship between ethnic identity and language attitudes among ethnic minority university students in Xinjiang [in Chinese]. Journal of Research on Education for Ethnic Minorities, 28(6), 65–69. https://doi.org/10.15946/j.cnki.1001-7178.2017.06.010 | Direct | Xinjiang; Ethnic minority university students | Quantitative descriptive; 4 universities; 1,048 valid questionnaires | Independent report |
| S06 | C157, C158 | Tang, L. (2016a). Associations among language attitudes, learning motivation, and ethnic identity among Mongolian university students [in Chinese]. Journal of Hubei Correspondence University, 29(24), 122–123; Tang, L. (2016b). Effects of English education on ethnic identity and language attitudes among Mongolian university students [in Chinese]. Journal of Hebei University of Engineering (Social Science Edition), 33(4), 104–107. | Direct | Gansu; Mongolian English-major students | Quantitative non-randomised; 30 students; single-group two-wave design | Same-dataset reports combined |
| S07 | QKCNKI018 | Hu, Q., & Dong, J. (2017). Ethnic identity, language proficiency, and language attitudes among Mongolian preparatory students [in Chinese]. Journal of Inner Mongolia Normal University (Philosophy and Social Sciences Edition), 46(4), 123–127. https://doi.org/10.3969/j.issn.1001-7623.2017.04.021 | Direct | Inner Mongolia; Mongolian preparatory students | Quantitative descriptive; 100 valid questionnaires | Independent report |
| S08 | C088 | Yang, M. (2018). Discourses on "authenticity": Language ideology, ethnic boundaries, and Tibetan identity on a multi-ethnic campus. Journal of Multilingual and Multicultural Development, 39(10), 925–940. https://doi.org/10.1080/01434632.2018.1458854 | Contextual | Beijing; Tibetan university students | Qualitative ethnography; 13-month ethnography; 86 Tibetan students plus teachers and peers | Independent report |
| S09 | C104 | Lhagyal, D. (2026). Diversity and ethnic integration in Chinese higher education: Insights from the experiences of indigenous Tibetans. Globalisation, Societies and Education. https://doi.org/10.1080/14767724.2026.2627959 | Contextual | Beijing; Tibetan university students | Critical ethnography; 15-month fieldwork; 43 Tibetan and 21 Han students | Independent report |
| S10 | C107 | Wang, H., & Chao, X. (2022). Language negotiation moments of ethnic Tibetan students in the People's Republic of China: An identity perspective. Language and Education, 36(4), 346–361. https://doi.org/10.1080/09500782.2021.1936545 | Contextual | University outside the principal minority regions; Tibetan university students | Qualitative multi-source study; 11 students; repeated interviews, WeChat contact, and observation, 2017 and 2020 | Independent report |
| S11 | C117 | Guo, X. G., & Gu, M. M. (2018). Reconfiguring Uyghurness in multilingualism: An internal migration perspective. Ethnic and Racial Studies, 41(11), 2028–2047. https://doi.org/10.1080/01419870.2017.1343485 | Contextual | Universities outside the principal minority regions; Uyghur migrant university students | Qualitative language ethnography; 13 students; interviews, focus groups, and follow-up | Independent report |
| S12 | C142 | Liu, T., Ma, S., Liu, Y., & Wu, X. (2021). Language attitudes in third-language acquisition among She university students in Fujian [in Chinese]. Journal of Anyang Institute of Technology, 20(3), 113–115. https://doi.org/10.19329/j.cnki.1673-2928.2021.03.030 | Contextual | Fujian; She university students | Mixed methods; 107 questionnaires plus 2 interviews | Independent report |
| S13 | C145 | Zhang, J., & Wei, L. (2020). Language use and attitudes among ethnic minority university students in the new-media environment [in Chinese]. Journal of Language and Literature, 40(6), 65–70. https://doi.org/10.3969/j.issn.1672-8610.2020.06.010 | Contextual | Guangxi; Ethnic minority university students | Quantitative descriptive; 202 valid online questionnaires | Independent report |
| S14 | C146 | Ling, S. (2020). Language use and attitudes among Dai university students [in Chinese]. Journal of Kunming Metallurgy College, 36(6), 70–75. https://doi.org/10.3969/j.issn.1009-0479.2020.06.013 | Contextual | Yunnan; Dai university students | Mixed methods; 81 Dai-language-major students; questionnaires and interviews | Independent report |
| S15 | C148 | Wang, Y. (2019). The motivational process of language behavior among ethnic minority university students [in Chinese]. Journal of Qujing Normal University, 38(5), 63–68. | Contextual | Yunnan; Ethnic minority university students | Mixed methods; 2 institutions; 903 valid questionnaires plus observation/interviews | Independent report |
| S16 | C154 | Wang, J. (2017). Language attitudes of Uyghur university students in Xinjiang [in Chinese]. Journal of Shaanxi Normal University (Philosophy and Social Sciences Edition), 46(4), 170–176. https://doi.org/10.15983/j.cnki.sxss.2017.0413 | Contextual | Xinjiang; Uyghur university students | Quantitative descriptive; 4 universities; 303 valid questionnaires | Independent report |
| S17 | C168 | Yang, Y. (2015). Language attitudes among ethnic minority university students in Yunnan in a trilingual context [in Chinese]. Guizhou Ethnic Studies, 36(8), 237–240. https://doi.org/10.13965/j.cnki.gzmzyj10026959.2015.08.058 | Contextual | Yunnan; Ethnic minority university students | Quantitative descriptive; 4 universities; 546 valid questionnaires | Independent report |
| S18 | C169 | Cui, Y., Zhang, L., & Du, L. (2015). Language attitudes of Uyghur university students and implications for language teaching [in Chinese]. Overseas English, (5), 62–63. | Contextual | Xinjiang; Uyghur university students | Quantitative descriptive; Tarim University; 106 valid questionnaires | Independent report |
| S19 | QKCNKI029, QKCNKI030 | Wang, Y. (2015a). Three-language cultural identification among ethnic minority university students [in Chinese]. Journal of Kunming Metallurgy College, 31(2), 42–46; Wang, Y. (2015b). Three-dimensional language–culture identification and second-language acquisition among ethnic minority vocational-college students [in Chinese]. Journal of Research on Education for Ethnic Minorities, 26(1), 109–113. | Contextual | Yunnan; Ethnic minority vocational-college students | Quantitative descriptive; 10 colleges; 1,118 valid questionnaires | Same-dataset reports combined |
| S20 | QKCNKI042 | Long, Y., & Duan, T. (2012). Cultural identity among Uyghur university students in Xinjiang [in Chinese]. Xinjiang Social Sciences, (6), 120–123. | Contextual | Xinjiang; Uyghur university students | Quantitative descriptive; 3 classes; 75 valid questionnaires | Partial overlap with S23 |
| S21 | C164 | He, L., & Wang, Y. (2016). Language attitudes and related factors among ethnic minority freshmen at science and engineering universities in Beijing [in Chinese]. Journal of Research on Education for Ethnic Minorities, 27(2), 94–102. https://doi.org/10.15946/j.cnki.1001-7178.2016.02.013 | Contextual | Beijing; Tibetan and Uyghur freshmen | Mixed methods; 41 freshmen; questionnaires, interviews, and observation | Independent report |
| S22 | QKCNKI015 | Lin, L., & Lan, Y. (2019). Language–culture identification among Zhuang-mother-tongue university students [in Chinese]. Education Teaching Forum, (6), 249–250. https://doi.org/10.20263/j.cnki.jyjxlt.2019.06.107 | Contextual | Guangxi; Zhuang-mother-tongue university students | Quantitative descriptive; 450 valid questionnaires | Independent report |
| S23 | QKCNKI048 | Long, Y., Oran, M., & Zhang, B. (2011). Language attitudes among Uyghur university students [in Chinese]. Journal of Xinjiang Normal University (Philosophy and Social Sciences Edition), 32(6), 102–108. https://doi.org/10.14100/j.cnki.65-1039/g4.2011.06.003 | Contextual | Xinjiang; Uyghur university students | Quantitative descriptive; 4 classes; 89 valid questionnaires | Partial overlap with S20 |

Note. A report is an individual publication; a study is the underlying empirical investigation or dataset. S01–S23 are continuous presentation identifiers for the 23 distinct studies. Original audit IDs are retained solely to link included reports to the review records; they do not indicate database source, evidence category, or study quality. Full references are also included in the manuscript reference list.

# Table S2. Study-level MMAT 2018 judgments

S1 and S2 are the screening questions; Q1–Q5 are the five design-specific criteria. Judgments are reported as Yes, No, or Can't tell. The final column records the main evidence or limitation that governed interpretation.

| **Study** | **Citation** | **Design** | **S1** | **S2** | **Q1** | **Q2** | **Q3** | **Q4** | **Q5** | **Evidence note / interpretive constraint** |
| --- | --- | --- | --- | --- | --- | --- | --- | --- | --- | --- |
| S01 | Liu (2026) | Qualitative | Yes | Yes | Yes | Yes | Yes | Yes | Yes | All design-specific criteria met. |
| S02 | Mao et al. (2026) | Mixed methods | Yes | Yes | Yes | Yes | Yes | Can't tell | Can't tell | Divergence: Can't tell; Component quality: Can't tell. |
| S03 | Kong (2023) | Quantitative descriptive | Yes | Yes | Yes | No | Can't tell | Can't tell | Yes | Representativeness: No; Measurement: Can't tell; Nonresponse: Can't tell. |
| S04 | Lin & Chen (2022) | Quantitative descriptive | Yes | Yes | Yes | Can't tell | Yes | Yes | Yes | Design category adjudicated because the qualitative component did not yield separately appraisable findings. Representativeness: Can't tell. |
| S05 | Ma (2017) | Quantitative descriptive | Yes | Yes | Yes | Yes | Yes | Yes | Yes | All design-specific criteria met. |
| S06 | Tang (2016a, 2016b) | Quantitative non-randomised | Yes | Yes | No | Can't tell | Yes | No | Can't tell | Representativeness: No; Measurement: Can't tell; Confounding: No; Exposure fidelity: Can't tell. |
| S07 | Hu & Dong (2017) | Quantitative descriptive | Yes | Yes | Yes | Can't tell | Yes | Yes | Yes | Representativeness: Can't tell. |
| S08 | Yang (2018) | Qualitative | Yes | Yes | Yes | Yes | Yes | Yes | Yes | All design-specific criteria met. |
| S09 | Lhagyal (2026) | Qualitative | Yes | Yes | Yes | Yes | Yes | Yes | Yes | All design-specific criteria met. |
| S10 | Wang & Chao (2022) | Qualitative | Yes | Yes | Yes | Yes | Yes | Yes | Yes | All design-specific criteria met. |
| S11 | Guo & Gu (2018) | Qualitative | Yes | Yes | Yes | Yes | Yes | Yes | Yes | All design-specific criteria met. |
| S12 | Liu et al. (2021) | Mixed methods | Yes | Yes | Yes | No | Can't tell | Can't tell | No | Integration: No; Interpretation: Can't tell; Divergence: Can't tell; Component quality: No. |
| S13 | Zhang & Wei (2020) | Quantitative descriptive | Yes | Yes | Yes | Can't tell | Can't tell | Yes | Yes | Representativeness: Can't tell; Measurement: Can't tell. |
| S14 | Ling (2020) | Mixed methods | Yes | Yes | Yes | Can't tell | Can't tell | Can't tell | Can't tell | Integration: Can't tell; Interpretation: Can't tell; Divergence: Can't tell; Component quality: Can't tell. |
| S15 | Wang (2019) | Mixed methods | Yes | Yes | Yes | Can't tell | Can't tell | Can't tell | Can't tell | Integration: Can't tell; Interpretation: Can't tell; Divergence: Can't tell; Component quality: Can't tell. |
| S16 | Wang (2017) | Quantitative descriptive | Yes | Yes | Can't tell | Can't tell | Can't tell | Yes | Yes | Sampling: Can't tell; Representativeness: Can't tell; Measurement: Can't tell. |
| S17 | Yang (2015) | Quantitative descriptive | Yes | Yes | Yes | Can't tell | Yes | Yes | Yes | Representativeness: Can't tell. |
| S18 | Cui et al. (2015) | Quantitative descriptive | Yes | Yes | Can't tell | Can't tell | Can't tell | Yes | Yes | Design category adjudicated because the qualitative component did not yield separately appraisable findings. Sampling: Can't tell; Representativeness: Can't tell; Measurement: Can't tell. |
| S19 | Wang (2015a, 2015b) | Quantitative descriptive | Yes | Yes | Yes | Yes | Yes | Yes | Yes | All design-specific criteria met. |
| S20 | Long & Duan (2012) | Quantitative descriptive | Yes | Yes | Yes | Can't tell | Can't tell | Yes | Yes | Representativeness: Can't tell; Measurement: Can't tell. |
| S21 | He & Wang (2016) | Mixed methods | Yes | Yes | Yes | Yes | Yes | Can't tell | No | Divergence: Can't tell; Component quality: No. |
| S22 | Lin & Lan (2019) | Quantitative descriptive | Yes | Yes | Yes | Can't tell | Can't tell | Yes | Yes | Representativeness: Can't tell; Measurement: Can't tell. |
| S23 | Long et al. (2011) | Quantitative descriptive | Yes | Yes | Yes | Can't tell | Can't tell | Yes | Yes | Representativeness: Can't tell; Measurement: Can't tell. |

# S3. Full-text exclusions

| **Record ID** | **Reason for exclusion** |
| --- | --- |
| C066 | Learner/multilingual identity; no eligible ethnic/cultural identity outcome. |
| QKCNKI011 | National common-language identification and national-community consciousness only. |
| QKCNKI038 | No identifiable sample, methods, instruments, or original empirical data. |
| C175 | Non-empirical conceptual discussion and ineligible identity construct. |
| QKCNKI046 | University and high-school participants combined; university findings not separately extractable. |

# S4. Search strategies

All searches were last run on 24 July 2026. CNKI was one database searched in two independent runs; Sections S4.3–S4.4 report the principal and supplementary runs separately. The strings below are transcribed from the retained native execution logs for Scopus, Web of Science, and CNKI and preserve the operational Boolean logic used to construct the 303-record pool.

## S4.1 Scopus (Elsevier; TITLE-ABS-KEY; n = 110)

TITLE-ABS-KEY((China OR Chinese OR Xinjiang OR Xizang OR Tibet* OR Yunnan OR Guangxi OR Qinghai OR Ningxia OR Guizhou OR "Inner Mongolia") AND ("ethnic minorit*" OR "minority group*" OR Uyghur* OR Uighur* OR Tibetan* OR Zhuang OR Mongol* OR Yi OR Kazakh* OR Kyrgyz* OR Hui OR Korean) AND (universit* OR college* OR undergraduate* OR "higher education" OR "tertiary education") AND (((language* OR linguistic* OR bilingual* OR multilingual* OR trilingual* OR Putonghua OR Mandarin OR English OR "mother tongue") AND ("ethnic identit*" OR "cultural identit*" OR "linguistic identit*" OR "language identit*" OR "national identit*" OR belonging OR subjectivit*)) OR "language attitude*" OR "linguistic attitude*" OR "language ideolog*" OR "language investment" OR "language maintenance" OR "heritage language*" OR "minority language*" OR "ethnic language*"))

## S4.2 Web of Science Core Collection (Clarivate; Topic; n = 70)

TS=((China OR Chinese OR Xinjiang OR Xizang OR Tibet* OR Yunnan OR Guangxi OR Qinghai OR Ningxia OR Guizhou OR "Inner Mongolia") AND ("ethnic minorit*" OR "minority group*" OR Uyghur* OR Uighur* OR Tibetan* OR Zhuang OR Mongol* OR Yi OR Kazakh* OR Kyrgyz* OR Hui OR Korean) AND (universit* OR college* OR undergraduate* OR "higher education" OR "tertiary education") AND (((language* OR linguistic* OR bilingual* OR multilingual* OR trilingual* OR Putonghua OR Mandarin OR English OR "mother tongue") AND ("ethnic identit*" OR "cultural identit*" OR "linguistic identit*" OR "language identit*" OR "national identit*" OR belonging OR subjectivit*)) OR "language attitude*" OR "linguistic attitude*" OR "language ideolog*" OR "language investment" OR "language maintenance" OR "heritage language*" OR "minority language*" OR "ethnic language*"))

## S4.3 Principal CNKI journal search (Reviewer 1; Topic; n = 55)

The principal CNKI journal search combined the same four Topic-field blocks as the supplementary search below, executed by Reviewer 1 using CNKI's advanced-search interface with Chinese-language equivalents of the English term blocks reported in S4.1–S4.2.

## S4.4 Independent supplementary search in CNKI (Reviewer 2; Topic; 68 journal records entering the pool)

Reviewer 2 used the CNKI advanced-search interface to combine four Topic-field blocks: (1) China and minority-region/ethnic-group terms; (2) university, college, undergraduate, postgraduate, preparatory, and higher-education terms; (3) language attitude, use, choice, ideology, bilingualism/multilingualism, Putonghua, English, mother tongue, and minority/heritage-language terms; and (4) ethnic, cultural, linguistic/language and culture identity and belonging terms. Sixty-eight journal records entered the combined pool. Thirty theses or dissertations retrieved by the same search were excluded before pool construction under the journal-only publication criterion.
